# The Pediatric Endocrinology Forum: Summer Camps for Diabetic Children in the Southeastern Regions of Turkey

**DOI:** 10.4274/Jcrpe.517

**Published:** 2012-03-08

**Authors:** Şükrü Hatun, Rüveyde Bundak, Mehmet Nuri Özbek

**Affiliations:** 1 Kocaeli University Department of Pediatric Endocrinology, Kocaeli, Turkey; 2 İstanbul University Faculty of Medicine, Department of Pediatric Endocrinology, İstanbul, Turkey; 3 Diyarbakır Children Hospital, Pediatric Endocrinology, Diyarbakir, Turkey; bruveyde@istanbul.edu.tr

**Keywords:** Diabetes, children, camps

## INTRODUCTION

Organizing and conducting a camp for diabetic children in a problematic region of Turkey has been a worthwhile and enriching experience for our team. The aim of this paper is to share this experience with our fellow pediatric endocrinologists around the world.

Summer camps for diabetic children serve many purposes. They aim to evaluate and improve patient knowledge on diabetes; to initiate experience- and communication-based learning in the diabetic children; and, most importantly, to help them acquire self-confidence and a realistic attitude towards their diabetic state. To accomplish these aims, the camp site needs to be well selected, the facilities - adequate and, most importantly, the team conducting the activities needs to be sufficient in number as well as in proficiency.

The first camp for diabetic children was conducted by Dr F.C.Wendt in the State of Michigan in 1925 (1). Today, over 350 camps are being held yearly around the world, addressing 15-20 000 diabetics (2). The immediate positive impact of the camp experience on metabolic control is well-documented. According to the experiences of Pediatric Endocrinology Unit at Akdeniz University, there was a significant decrease in HbA1c levels 6-12 months after the camp and the level of self-care increased (3,4). Even more important is the motivation, which the children gained from the camp experience, to lead a good life in peace with the diabetic state. The first camp for diabetic children in Turkey was organized in 1993 by the Pediatric Endocrinology Unit of the Istanbul Faculty of Medicine. Antalya, a city in southern Turkey, was selected as the first camp site. Following this first venture, with the support of the University administration and the sponsorship of a major firm in the private sector, the Istanbul team has been able to continue this endeavor each summer, and approximately 100 children benefit from the camp for one week each year. In recent years, several Pediatric Endocrine Units around the country have taken an interest in this educational activity for the diabetic children attending their clinics, and several camps for diabetic children are being held each summer. The efforts of Kocaeli University in continuing and extending this activity to less developed regions in Turkey also need to be noted.

The Eastern and Southeastern regions of Turkey continue to be the least developed areas of the country. In addition to problems created by socioeconomic conditions, low level of literacy, ethnic diversity, and communication difficulties also hinder easy access to the population in certain pockets in the region. The first camp for diabetic patients in Eastern/Southeastern Turkey was conducted in 2002 in Elazig, a major city in the region, with the joint efforts of the Turkish Diabetes Foundation and the Pediatric Endocrine Unit at Kocaeli University and with the participation of adult endocrine units in the region. This activity was continued yearly for 3 consecutive years. Diabetic camps were also organized in another city of the region in 2003 and 2004.

In recent years, organization of camps for diabetic children was listed as one of the 6 major activities in enhancing diabetic care by the National Program for the Control of Diabetes. This statement was an important step in motivating educational activities in pediatric diabetic care. 

A recent 5-day camp activity for diabetic children was held in a site on the border of a lake near Diyarbakir, a major city in Southeastern Turkey, in August 2011 and was organized and conducted by the Endocrine Clinic of the Pediatric Department at the Diyarbakir State Hospital, with the support of the Diabetes Group at the Turkish Pediatric Endocrinology and Diabetes Society, the Turkish Pediatric and Adolescent Diabetes Association and the Endocrine Units at Kocaeli and Istanbul Universities.

The 56 children and adolescents who attended the camp were between 10 and 18 years and came from 19 cities and smaller settlements in the region. One of these children was a girl who never had had a chance to attend school and who understood and spoke only Kurdish. The team conducting the training and other activities of the camp included 26 health workers from various provinces of Turkey and 11 health workers from the Diyarbakir municipality. In the educational sessions done by the experts in small groups 2 hours a day, different aspects of diabetes were discussed including details of insulin treatment, signs of hypoglycemia/hyperglycemia and their treatment, and importance of exercise. At meal times and during the sport sessions, application education was performed. Meal planning and carbohydrate counting were explained by a dietician and problem solving was done at each meal. Group therapy was conducted by a psychologist. Local and national media were invited to the camp; interviews aimed at increasing the awareness about the diabetes disease were given.

The pediatric population in Southeastern Turkey is estimated to be 2 882 114 and of these, 1900 are presently being followed in various health institutions with a diagnosis of diabetes mellitus. The number of diabetic children who regularly attend the State Hospital in Diyarbakir is 450. Although the quality of diabetes care has improved greatly in recent years, there is still more to be done, particularly regarding the education of the diabetic child and his/her family. We believe that the camp activity conducted in August has contributed to diabetic care in the region. Moreover, Diyarbakir Diabetic Summer Camp has increased the knowledge and skills of diabetic children about their disease and also has given the medical staff a chance to get to know their patients better and closer and has created a motivation in all people involved in the follow-up process.

Another end product of this camp was the decision to conduct a collaborative project by a team in a city in the Western part of Turkey (Kocaeli) and a team in Diyarbakir. We hope to continue to organize summer camps for diabetic children in this region of Turkey in the future, with the collaboration of our pediatric colleagues working in the region.

**Şükrü Hatun, MD, Professor of Pediatrics, Kocaeli University**

**Rüveyde Bundak, MD, Professor of Pediatrics, Istanbul University**

**Mehmet Nuri Özbek, MD, Asst. Professor of Pediatrics, Diyarbakir State Children Hospital**

**Hüseyin Demirbilek MD, Pediatric Endocrinologist, Diyarbakir State Children Hospital**

**Filiz Çizmecioğlu Asst. Professor of Pediatrics, Kocaeli University**

## References

[1] American Diabetes Association (2007). Diabetes care at diabetes camps. Diabetes Care.

[2] McAuliffe-Fogarty AH, Ramsing R, Hill E (2007). Medical specialty camps for youth with diabetes. Child Adolesc Psychiatr Clin N Am.

[3] Semiz S, Bilgin UO, Bundak R, Bircan I (2000). Summer camps for diabetic children: an experience in Antalya, Turkey. Acta Diabetol.

[4] Karaguzel G, Bircan I, Erisir S, Bundak R (2005). Metabolic control and educational status in children with type 1 diabetes: effects of a summer camp and intensive insulin treatment. Acta Diabetol.

